# The application of a new type of intestinal diversion devices in Beagle dogs with intestinal transection injury and hemorrhagic shock

**DOI:** 10.3389/fbioe.2026.1772513

**Published:** 2026-05-08

**Authors:** Ridong Zhou, Jichang Zheng, Gongxin Yang, Jing Tu, Meiyu Chen, Hu Zhao, Yu Wang, Xiaobin Chen, Chen Lin

**Affiliations:** 1 Department of General Surgery, Fuzong Clinical Medical College of Fujian Medical University, Fuzhou, Fujian, China; 2 Department of General Surgery, 900th Hospital of PLA Joint Logistic Support Force, Fuzhou, Fujian, China; 3 Department of General Surgery, Fuzong Teaching Hospital of Fujian University of Traditional Chinese Medicine, (900th Hospital), Fuzhou, Fujian, China; 4 Department of General Surgery, Dongfang Hospital of Xiamen University, School of Medicine, Xiamen University, Fuzhou, Fujian, China

**Keywords:** damage control surgery, injury necrosis, intestinal diversion, intestinal injury, intestinal diversion device

## Abstract

**Objective:**

This study evaluated the efficacy and safety of a series of prototype intestinal diversion devices in a Beagle hemorrhagic shock model comprising four complex bowel-injury patterns (small and large bowel).

**Methods:**

Sixteen Beagle dogs were randomized to four intestinal injury models (n = 4 each): (1) single-site small bowel transection, (2) suspected long-segment small-bowel ischemia/necrosis, (3) two-site small bowel transection, and (4) combined small-bowel injury with colon rupture. Within each cohort, animals were allocated to either conventional suture ligation of the bowel ends (bowel left in discontinuity) or device-mediated diversion to temporarily restore luminal continuity. Systemic inflammation (blood pH, endotoxin, TNF-α, IL-6, IL-10) was monitored at baseline and 2, 4, 8, and 24 h postoperatively. On postoperative day 30, tissue samples from the intestinal stump were excised to evaluate morphological changes and the extent of inflammatory infiltration via light microscopy.

**Results:**

Baseline characteristics were comparable between groups, and all animals (16/16) survived to day 30. Postoperatively, endotoxin, TNF-α, and IL-6 increased in both groups but were markedly attenuated in the diversion group. IL-10 showed a higher increase in the diversion group. The length of necrotic bowel requiring resection was consistently shorter with diversion. On postoperative day 30, histology showed well-perfused anastomoses with mild inflammation in the diversion group, whereas the ligation group exhibited ischemic necrosis with severe transmural inflammation.

**Conclusion:**

Preliminary findings suggest that the new intestinal diversion devices represent a feasible and promising strategy for damage control in complex intestinal trauma, particularly in the setting of combined injuries and hemorrhagic shock.

## 1 Introduction

In critically ill patients with gastrointestinal injuries, primary repair is often not feasible due to hypothermia, acidosis, and coagulopathy (Richman and Burlew, 2019; Petros, 2019; Iba et al., 2023; Bonanno, 2023). Damage control surgery (DCS) has emerged as a surgical strategy aimed at rapidly restoring physiological stability and has significantly reduced mortality among such patients (Cirocchi et al., 2021; Chung and Scalea, 2023). The DCS approach typically consists of three stages: (1) an initial abbreviated operation to control bleeding and contamination; (2) a resuscitative phase to correct physiological derangements; and (3) a definitive surgical phase for anatomical reconstruction once the patient’s condition stabilizes (Chung and Scalea, 2023; Fernandez, 2023). Conventionally, intestinal damage control dictates the resection of injured segments and ligation of bowel ends to rapidly achieve source control (Ye et al., 2024; He et al., 2024), with restoration of continuity deferred to a planned re-exploration within 12–48 h (Serfin et al., 2024). However, while rapid ligation or stapling achieves immediate source control during the damage-control phase, it inherently leaves the bowel in discontinuity, creating a functionally obstructed segment. This ‘blind-ended’ configuration can exacerbate intraluminal hypertension and impair microvascular perfusion, thereby predisposing the bowel to secondary ischemic necrosis and propagating inflammation during the interval prior to definitive reconstruction (Littlefield et al., 2025; Ellis and House, 2024; Mullen et al., 2020). Therefore, a rapid, non-anastomotic strategy that maintains temporary luminal patency and allows for decompression could preserve intestinal viability, thereby optimizing the substrate for definitive second-stage reconstruction. In this study, we evaluated a novel intestinal diversion device—fabricated from biocompatible silicone to ensure flexibility and safety—in a canine model of combined enterocolonic injury complicated by hemodynamic shock. By benchmarking this device against conventional bowel ligation, we aimed to identify a more physiological and feasible strategy for damage control in complex intestinal trauma.

## 2 Materials and methods

### 2.1 New type of intestinal diversion devices

**FIGURE 1:**
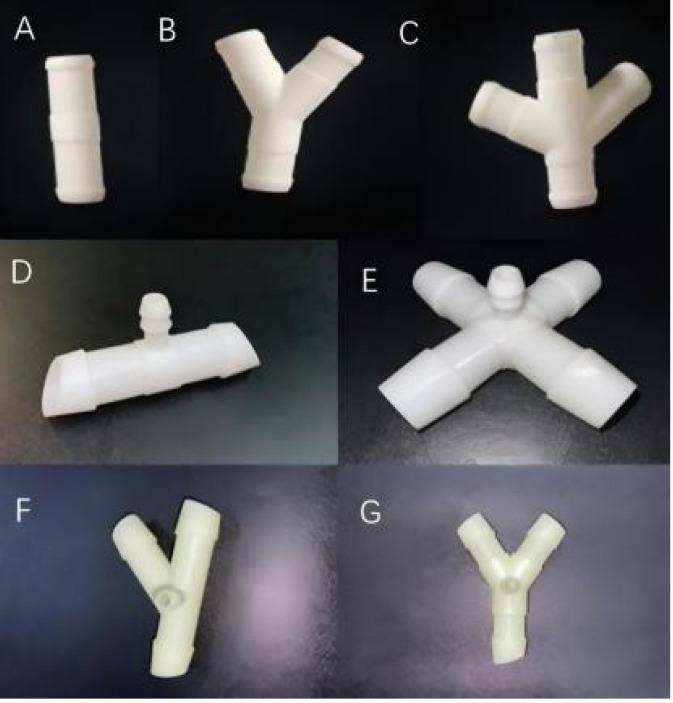
Schematic representation of the new intestinal diversion devices. **(A,D)** straight-tube type diversion device; **(B,G)** Y-shaped diversion device; **(C,E)** Four-branch diversion device; **(F)** Three-branch diversion device.

A series of prototype intestinal diversion devices was fabricated from medical-grade addition-cure liquid silicone rubber. A predetermined quantity of the compound was loaded into a mold cavity, which was then sealed, heated to 130 °C, and maintained at a pressure of 15 MPa for 20 min to ensure complete vulcanization and cross-linking. After cooling and demolding, the outer surface of each connector device was post-processed to create a helical texture for enhanced frictional stability upon insertion. The main conduit was designed with an outer diameter of 12–16 mm, an inner diameter of 10–12 mm, and a wall thickness of approximately 2.5 mm. To accommodate larger bowel segments, specific variants were produced with an inner diameter of 20 mm. Each connection port featured an oblique-cut profile, and annular grooves were incorporated along the edges to optimize sealing and security. Depending on the clinical requirements, the system allowed for various configurations, including straight, Y-shaped, three-branch, and four-branch architectures. Optional integrated drainage tubes, with or without lateral apertures, were included to facilitate effluent management and bowel decompression. Following fabrication, all devices were sterilized using ethylene oxide (EtO) and stored under sterile conditions until use (Figure 1).

### 2.2 Experimental animals

A total of 16 healthy male Beagles aged 6 months were obtained from Fuzhou Zhenhe Experimental Animal Technology Development Co., Ltd. (License No.: SCXK [Min] 2018-0001). The Beagles were housed in standardized facilities with environmental temperatures maintained at 26 °C–28 °C and relative humidity at 60%–80%. They were allowed free access to food and water. Routine monitoring of the animals’ overall condition was performed, and perioperative pain levels, food intake, activity levels, and wound status were assessed. After confirming that all Beagles had no underlying diseases or adverse conditions, they were randomly divided into four injury model groups using a random number table. Subsequently, they were further allocated to the ligation group and diversion group: (1) single-site small bowel transection, (2) suspected long-segment small-bowel ischemia/necrosis, (3) two-site small bowel transection, and (4) combined small-bowel injury with colon rupture. Each major group was further stratified into two subgroups (n = 2 per subgroup): a ligation subgroup, in which intestinal ends were subjected to conventional suture ligation to interrupt continuity, and a diversion subgroup, in which intestinal continuity was re-established using the new silicone diversion device. All experimental procedures were conducted in strict accordance with the guidelines of the Animal Ethics Committee of the People’s Republic of China for the care and use of laboratory animals and relevant national regulations. The study protocol was reviewed and approved by the Ethics Committee of the 900th Hospital of the PLA Joint Logistic Support Force (Approval No. 2024-019).

### 2.3 Experimental method

#### 2.3.1 Construction of hemorrhagic shock model

Anesthesia was induced via intramuscular injection of xylazine hydrochloride at a dose of 0.1–0.2 mL/kg. If adequate immobilization was not achieved due to individual variation, an additional 25%–33% of the initial dose was administered. After anesthesia onset, each Beagle dog was positioned on the operating table. An arterial indwelling catheter was inserted into the left femoral artery, and a venous indwelling catheter was placed in the right femoral vein. Controlled hemorrhage was then performed via the femoral arterial catheter to induce hemorrhagic shock. Blood was withdrawn over a period of 20 min, with a total volume equivalent to approximately 40% of the estimated total blood volume (about 35 mL/kg). The shock state was maintained for 30 min without fluid resuscitation to establish a stable hemorrhagic shock model, simulating the time interval between injury and effective medical intervention.

#### 2.3.2 Establishment of different intestinal injury models

**FIGURE 2:**
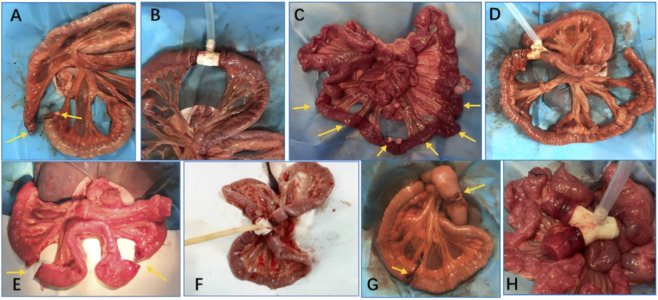
Establishment of intestinal injury models. **(A)** Single-site small intestinal injury model; **(B)** Straight-tube intestinal diversion device model; **(C)** Long-segment intestinal suspected necrosis model; **(D)** Three-branch diversion device model; **(E)** Two-site Small Bowel Transection injury model; **(F)** Four-branch diversion device model; **(G)** Single Small Bowel Injury Combined with Colon Rupture model; **(H)** Y-shaped diversion device model.

Under deep general anesthesia, a midline laparotomy was performed to access the abdominal cavity and expose the target intestinal segment for model creation. Single-site small bowel transection model: A single small bowel transection model was established by transecting the intestine 55 cm distal to the Treitz ligament. Thirty minutes after the induction of hemorrhagic shock, the severed bowel ends in the ligation group were closed using conventional suture ligation (Figure 2A). In contrast, the diversion group received the straight-tube type diversion device to rapidly reconstruct the bowel (Figure 2B). Suspected long-segment ischemia/necrosis model: A 50-cm segment of the small intestine was isolated, beginning 55 cm distal to the Treitz ligament. Within this segment, five segments with impaired blood supply and five with normal blood supply were established, separated by 5-cm intervals. Ischemia was induced by ligating the main supplying vessels while preserving collateral branches; this compromised blood flow and induced potential necrosis, but maintained bowel wall integrity without rupture (Figure 2C). After 30 min of hemorrhagic shock, the proximal end of the ischemic segment was ligated in the ligation group, whereas the diversion group transected the bowel at both ends of the 50-cm ischemic segment. The suspected necrotic segment was ligated at the proximal end, and the distal end was diverted via the branches of the three-branch diversion device, with the two intact ends connected to the main body of the device. A drainage tube was placed to collect and monitor intestinal effluent, thereby facilitating bowel decompression (Figure 2D). Two-site small bowel transection model: Small bowel transections were performed at 55 cm and 120 cm distal to the Treitz ligament to create a two-site injury model (Figure 2E). Following 30 min of hemorrhagic shock, the diversion group utilized the four-branch diversion device to rapidly restore intestinal continuity by connecting the four transected bowel ends. Integrated drainage tubes were attached to facilitate decompression (Figure 2F), whereas the ligation group closed each bowel end by conventional suture ligation, leaving the bowel in discontinuity. Combined small-bowel injury with colon rupture model: A single-site small bowel transection was performed, and a concomitant colonic perforation was created (Figure 2G). Following 30 min of hemorrhagic shock, the diversion group utilized a Y-shaped diversion device to reconstruct the two transected ends of the small bowel and the colonic rupture site (Figure 2H), whereas the ligation group underwent conventional suture ligation of the bowel ends without restoring luminal continuity. Following the completion of the respective intestinal transection, diversion, or ligation procedures, the peritoneal cavity was lavaged with sterile saline, and the abdomen was closed in layers. Subsequently, controlled hypotensive resuscitation was initiated with 0.9% sodium chloride, targeting a systolic blood pressure >80 mmHg or a mean arterial pressure >60 mmHg. Prior to abdominal closure, an ultrasound-guided abdominal wall nerve block was performed using 0.25% lidocaine (prepared by diluting 2% lidocaine with saline at a 1:7 ratio) to provide sensory blockade. Subsequently, postoperative local infiltration analgesia was administered at the incision site using 0.5% lidocaine (diluted 1:3 with saline). All Beagles underwent a second laparotomy 24 h after the initial procedure to perform definitive intestinal reconstruction and to assess the morphology of the abdominal cavity and anastomotic sites. All experimental Beagles were euthanized and examined at 30 days postoperatively. Animals were placed in a purpose-built euthanasia chamber and exposed to 100% carbon dioxide (CO_2_) introduced using a gradual-fill (displacement) method at a controlled flow rate equivalent to 40% of the chamber volume per minute. CO_2_ delivery was regulated via a pressure-reducing regulator and flow meter and animals were continuously observed throughout the procedure. CO_2_ administration was continued until respiratory arrest and was maintained for ≥1 min after apnea. Death was confirmed by the absence of cardiac activity on auscultation and loss of corneal/pedal withdrawal reflexes prior to necropsy and tissue collection.

### 2.4 Data collection and analysis

Serial arterial and venous blood samples were obtained from the femoral vessels at baseline (0 h) and at 2, 4, 8, and 24 h postoperatively. Arterial samples were analyzed for blood pH, while venous plasma was assayed for endotoxin, TNF-α, IL-6, and IL-10 using ELISA. A planned second-look laparotomy was performed 24 h after the initial damage control surgery to achieve definitive intestinal reconstruction. Following recovery from anesthesia, all animals received standard postoperative care and were monitored for vital sign stability. Survival was tracked daily until the study endpoint on postoperative day 30. Clinical assessments included food intake, general activity, and signs of abdominal distension. Gastrointestinal function was evaluated based on defecation frequency, stool consistency (including the presence of diarrhea or constipation), and abdominal auscultation to detect potential dysmotility or obstruction. These functional outcomes were recorded qualitatively as part of the routine follow-up protocol. On day 30, the animals were euthanized, and tissue samples were collected. The samples were fixed in 10% neutral buffered formalin for 24 h, embedded in paraffin, and sectioned. They were then stained with hematoxylin and eosin (H&E) for histopathological examination under a light microscope.

### 2.5 Statistical methods

Statistical analyses were performed using SPSS 26.0 and GraphPad Prism 8.4. Continuous variables with a normal distribution were expressed as mean ± standard deviation (SD) and compared using the independent Student’s t-test. Non-normally distributed data were presented as median [IQR] and analyzed via the Mann-Whitney U test. For subgroup analyses with limited sample sizes (n = 2), data were reported descriptively as means. A P-value <0.05 was considered statistically significant.

## 3 Results

### 3.1 Baseline characteristics and perioperative outcomes

**FIGURE 3:**
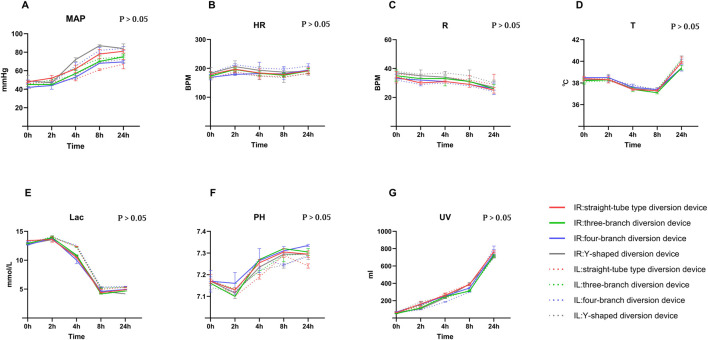
Temporal trends in physiological and metabolic parameters over the 24-h perioperative period. The line graphs illustrate the longitudinal trajectories of **(A)** Mean Arterial Pressure (MAP), **(B)** Heart Rate (HR), **(C)** Respiratory Rate (R), **(D)** Body Temperature (T), **(E)** Lactate (Lac), **(F)** PH, and **(G)** Urine Volume (UV) at 0, 2, 4, 8, and 24 h. In all panels, the solid line (IR) represents the diversion group, while the dashed line (IL) denotes the ligation group.

There were no statistically significant differences between the diversion and ligation groups in terms of baseline characteristics, blood loss, or operative time (P > 0.05; Table 1). Furthermore, serial monitoring of vital signs and metabolic parameters demonstrated sustained recovery across all groups post-resuscitation. The diversion group exhibited a recovery profile comparable to the ligation group. As shown in Figure 3, there were no significant inter-group differences in MAP, heart rate (HR), respiratory rate (R), body temperature (T), lactate (Lac), PH, and urine volume (UV) at any postoperative time point (0–24 h). These findings indicated that the implantation of the diversion device did not induce additional hemodynamic instability or metabolic disturbance compared to standard ligation. Throughout the study period, no adverse events, such as suture dehiscence or device dislodgement, were observed. No intraoperative or postoperative mortality was observed in either group. All animals successfully underwent the planned second-look laparotomy at 24 h and survived to the study endpoint on postoperative day 30, resulting in a 100% survival rate (16/16) for both the diversion and ligation groups.

**TABLE 1:** Comparison of weight, PH, blood volume, resuscitation fluid volume, and time required for intestinal stump management between the two groups.

Target	Diversion group	Ligation group	*P* value
Weight (kg)	14.75 ± 0.42	14.78 ± 0.57	>0.05
PH	7.23 ± 0.09	7.21 ± 0.15	>0.05
Blood volume (mL)	217.50 ± 8.06	224.50 ± 16.54	>0.05
Resuscitation fluid volume (mL)	785.00 ± 26.46	792.50 ± 29.86	>0.05
Time required for intestinal stump management (min)	5.37 ± 0.75	5.00 ± 0.82	>0.05

### 3.2 Inflammatory markers and endotoxin levels

**FIGURE 4:**
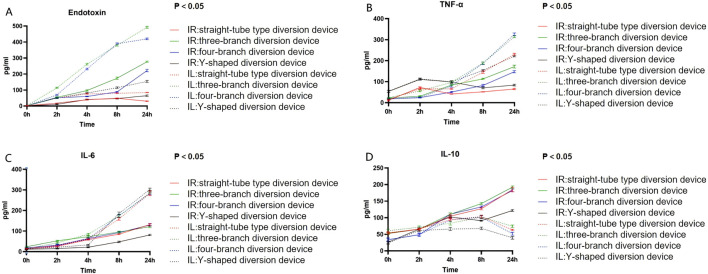
24-h changes in inflammatory markers and endotoxin (solid line IR: diversion group; dashed line IL: ligation group; colors indicate different injury models). **(A)** Endotoxin; **(B)** TNF-α; **(C)** IL-6; **(D)** IL-10.

Endotoxin, TNF-α, and IL-6 levels exhibited a time-dependent elevation in both the ligation and diversion groups over the observation period. Notably, the ligation group demonstrated a markedly more pronounced surge in these pro-inflammatory markers compared to the diversion group. Conversely, while IL-10 levels also increased in both groups, the diversion group mounted a substantially more robust anti-inflammatory response, evidenced by markedly higher IL-10 concentrations compared to the ligation group (Figure 4).

### 3.3 Length of necrotic intestine

The length of necrotic bowel resected was markedly shorter in the diversion group compared to the ligation group (Table 2).

**TABLE 2:** Length of necrotic intestinal segment resected before anastomosis in the two groups.

Type of device	Diversion group	Ligation group	*P* value
Straight-tube type diversion device (cm)	2.25	4.25	P < 0.05
Three-branch diversion device (cm)	20.50	35.50	P < 0.05
Four-branch diversion device (cm)	3.00	5.25	P < 0.05
Y-shaped diversion device (cm)	2.50	5.25	P < 0.05

### 3.4 Postoperative clinical course and gastrointestinal recovery

Following definitive reconstruction at 24 h, the diversion group maintained normal defecation patterns throughout the follow-up period (up to POD 30). Stools were predominantly well-formed, with only intermittent episodes of diarrhea and no clinical signs of constipation or obstruction. In contrast, despite identical dietary protocols, the ligation group frequently exhibited diarrhea of varying severity, particularly during the first postoperative week. Auscultation in this group often revealed markedly hyperactive bowel sounds. Qualitatively, the diversion group demonstrated improved appetite and general body condition compared to the ligation group. However, these parameters were assessed qualitatively as descriptive observations and were not subjected to formal statistical analysis in the present study.

### 3.5 Intestinal morphology

**FIGURE 5:**
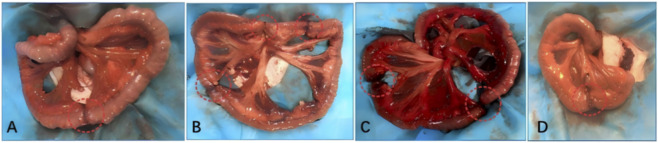
**(A)** In the straight-tube type diversion device group, the anastomotic site appeared grossly viable with good blood perfusion, minimal exudation, and no obvious ischemic necrosis. **(B)** In the three-branch diversion group, all anastomotic sites showed adequate blood supply with only limited local necrosis; the intestinal mucosa appeared reddish and viable. **(C)** In the four-branch diversion device group, the intestinal segments were grossly viable with homogeneous perfusion, showing no apparent necrosis or exudation. Upon opening the anastomotic site, the inner mucosal surface showed no evidence of localized necrosis, and only minimal necrotic tissue was observed. **(D)** In the Y-shaped diversion group, the intestinal wall appeared uniformly reddish, and both the small intestinal anastomosis and colonic stoma exhibited good morphology and perfusion.

During the 24-h second-look laparotomy, no significant adhesions were observed in either group. However, the ligation group exhibited extensive necrosis and discoloration at the transected ends, whereas the diversion group showed only mild ischemic changes. Additionally, while peritoneal effusion was present in both groups, it was notably more pronounced in the ligation group. Upon gross examination at day 30, the diversion group demonstrated well-perfused intestinal segments with preserved morphology. The anastomotic sites appeared viable (Figure 5), and the length of resected necrotic bowel was significantly shorter than that in the ligation group (Table 2). Conversely, the ligation group displayed marked ischemic necrosis at the anastomotic sites, accompanied by substantial fluid accumulation. Notably, despite the resection of necrotic tissue and re-anastomosis performed at 24 h, ischemic changes persisted at the anastomotic sites in the ligation group.

### 3.6 Histopathological comparison

**FIGURE 6:**
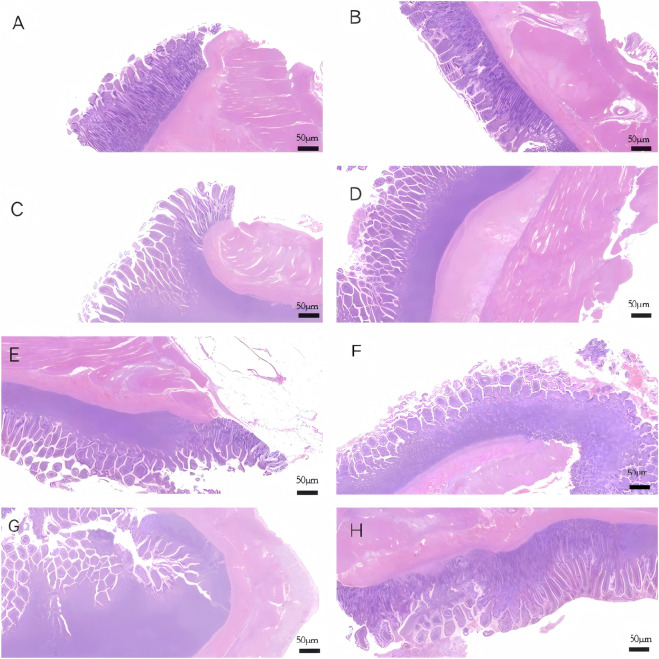
Histological morphology of the intestinal mucosa in different types of new diversion device and corresponding ligation groups (H&E staining, ×200). **(A)** Straight-tube type diversion group; **(B)** Straight-tube type ligation group; **(C)** Three-branch diversion group; **(D)** Three-branch ligation group; **(E)** Four-branch diversion group; **(F)** Four-branch ligation group; **(G)** Y-shaped diversion group; **(H)** Y-shaped ligation group.

Histological examination via light microscopy revealed distinct morphological differences between groups. In the diversion group, the segment 0.5 cm proximal to the transection site exhibited only mild inflammatory cell infiltration confined to the mucosa. In contrast, tissue within 0.5 cm of the ligation site in the ligation group demonstrated severe epithelial necrosis and mucosal sloughing, accompanied by extensive transmural inflammatory infiltration (Figure 6).

## 4 Discussion

In combat settings, penetrating gunshot wounds and complex abdominal injuries are frequently complicated by severe hemorrhagic shock (Singh et al., 2023; Bickel et al., 2022). This physiological insult often precipitates the ‘lethal triad’ of hypothermia, coagulopathy, and acidosis, culminating in systemic organ dysfunction and failure (Kyle et al., 2024; Moore et al., 2021; Dupuy et al., 2025). Attempting complex, prolonged primary repair under such critical conditions imposes a severe physiological burden, exacerbating secondary injury and resulting in increased morbidity and mortality (Surlin, 2021). In contemporary damage control surgery for complex bowel trauma, temporary ligation of transected ends is a standard strategy to rapidly arrest contamination (Chung and Scalea, 2023). Although effective in controlling intra-abdominal spillage and hemorrhage, this method inevitably creates intestinal discontinuity and closed-loop obstruction. Consequently, significant fluid sequestration and severe intestinal edema occur during the resuscitation phase, potentially precipitating hemodynamic instability (Willms et al., 2022). Moreover, the resulting closed-loop obstruction significantly elevates intraluminal pressure. This pathological state potentiates bacterial and endotoxin translocation due to gut barrier failure and precipitates abdominal compartment syndrome (ACS). Collectively, these insults fuel systemic inflammation and metabolic derangement, ultimately propelling the progression to multiple organ dysfunction syndrome (MODS) (Maitz et al., 2021). In contrast, our device serves as a temporary bridge between the initial damage-control operation and the planned definitive reconstruction. It permits rapid deployment, obviating the need for a formal primary anastomosis, while simultaneously restoring luminal continuity and enabling controlled decompression (facilitating drainage as required). This critical distinction allows the device to maintain patency and enable decompression, thereby mitigating pressure-induced secondary injury (distension, edema, ischemia) and attenuating the local inflammatory cascade—which collectively optimize tissue viability for subsequent reconstruction. Therefore, we advocate the use of this novel intestinal diversion device in scenarios of severe combat trauma or anticipated delayed evacuation. This strategy circumvents the need for primary anastomosis while preserving intestinal continuity and facilitating external drainage of contents when necessary. Crucially, it not only mitigates intraluminal hypertension but also enables real-time monitoring of intestinal effluent, facilitating the early detection of pathological changes.

Regarding hemodynamic and metabolic stability, time-course monitoring revealed comparable recovery profiles in both groups following resuscitation, with no significant inter-group differences observed (Figure 3). This finding is clinically pivotal for two reasons. First, it validates the intraoperative safety of the diversion strategy, confirming that device deployment did not prolong operative stress or induce additional hemodynamic instability compared to simple ligation. Second, despite the marked reduction in intestinal necrosis observed in the diversion group (Table 2), no corresponding differences were detected in systemic pH and lactate levels. This highlights the limited sensitivity of these markers in detecting segmental ischemia during massive resuscitation. Aggressive volume replacement likely improved global perfusion and induced a ‘washout effect,’ thereby masking the local metabolic acidosis generated by the closed-loop obstruction in the ligation group. Furthermore, the use of 0.9% sodium chloride may have contributed to hyperchloremic metabolic acidosis across all subjects (Fenves and Allegretti, 2025), narrowing the pH differential between groups. Thus, while systemic parameters suggested comparable physiological stability, local tissue outcomes underscored the superior protective efficacy of the diversion device.

Silicone is commonly used as a biomaterial for implants and catheters in the medical field (Marmo and Grunlan, 2023). Previous studies have highlighted the immunomodulatory properties of silicone (Barthes et al., 2021), particularly its capacity to suppress the upregulation of pro-inflammatory cytokines such as TNF-α and IL-6 (Kim et al., 2021). In this study, silicone was selected as the primary biomaterial for the fabrication of four novel intestinal diversion device prototypes. Endotoxin, TNF-α, and IL-6 were selected as key markers due to their synergistic roles in amplifying the inflammatory cascade (Yu et al., 2022). Specifically, TNF-α upregulates IL-6 expression (Xu et al., 2022; Miller et al., 2019), while IL-6 reciprocally amplifies TNF-α signaling (Saribal et al., 2019; Gao et al., 2017). This positive feedback loop compromises mucosal integrity, thereby facilitating bacterial and endotoxin translocation and perpetuating the systemic inflammatory response (Nishimura et al., 2021; Ling et al., 2022; Kaewduangduen et al., 2022). Our results indicate that the diversion group exhibited a marked attenuation of TNF-α and IL-6 levels compared to the ligation group, accompanied by a substantial upregulation of IL-10. Macroscopically, intestines in the ligation group were severely edematous, discolored, and ischemic, presenting with varying degrees of necrosis ranging from focal to transmural infarction. Histopathological analysis confirmed severe mucosal epithelial necrosis and sloughing in the ligation group, with extensive transmural inflammatory cell infiltration. In sharp contrast, the diversion group displayed only mild inflammatory infiltration confined to the mucosa, preserving structural integrity. These macroscopic and microscopic findings corroborate the core physiological advantages of diversion over ligation. The observed attenuation of the systemic inflammatory response, including cytokine modulation, is therefore primarily ascribed to the successful surgical decompression and maintenance of intestinal continuity achieved by the diversion procedure itself. While the clinical benefits are predominantly attributable to this surgical strategy, the specific contribution of the silicone material to local inflammatory modulation warrants further investigation. We hypothesize that the smooth surface of the silicone conduit minimizes mechanical irritation at the anastomotic site, thereby mitigating local stasis and mechanical stress. To definitively isolate the material-specific effects, future controlled studies comparing silicone devices with those composed of alternative biocompatible polymers (e.g., polyurethane) in identical injury models are essential. Furthermore, surface functionalization could be employed to actively optimize biocompatibility, potentially fine-tuning the local inflammatory microenvironment by modulating protein adsorption and immune cell interactions.

In this study, four distinct configurations of intestinal diversion devices were engineered. Specifically, the structural design of these devices features a proximal retention groove that ensures secure fixation, significantly reducing the risk of device migration or dislodgement. Furthermore, the beveled design at both termini facilitates streamlined insertion and optimizes flow dynamics, thereby minimizing operative time and mitigating mechanical trauma to the intestinal mucosa. The straight-tube type diversion device is specifically designed for single-site intestinal damage. In cases of a single rupture, the proximal and distal ends of the intestine can be expeditiously connected to the straight-tube type diversion device, thereby enabling rapid intestinal reconstruction. The three-branch diversion device is designed when a long-segment of the intestine is suspected to be necrotic. The proximal and distal ends of this segment are first disconnected. The proximal stump is ligated, and the distal end is connected to one branch of the device. The remaining two branches are utilized to connect the healthy intestinal segments. Following a 24–48 h observation period, a second-look evaluation is performed. If the segment suspected to be necrotic exhibits signs of irreversible ischemia (e.g., congestion, necrosis, or compromised perfusion), it is excised; otherwise, it is preserved and reconnected. Compared to traditional methods, this approach maintains intestinal continuity while minimizing the risk of short bowel syndrome caused by unnecessary resections. The four-branch diversion device is specifically designed for complex cases involving combined damage to two small intestine segments and two colon segments. By utilizing its four distinct ports, the device facilitates the expeditious restoration of continuity across these ruptured segments. This rapid deployment significantly minimizes visceral exposure time and enhances the overall viability of the compromised intestine. Finally, the Y-shaped diversion device is specifically indicated for single-site small intestine injury combined with concomitant colon perforation. In this configuration, the two branches of the Y-shaped device are connected to the proximal and distal ends of the small intestine to restore its continuity, while the main stem is utilized to manage the colon perforation. This approach facilitates a rapid diversion, effectively maintaining small intestinal continuity while simultaneously addressing the colonic defect.

Aligned with the tenets of damage control resuscitation, this study aims to refine trauma management strategies through the development of a new intestinal diversion device. This innovation represents a paradigm shift in the acute management of severe abdominal trauma, transitioning from traditional simple ligation to temporary intestinal reconstruction. The successful application of this rapid diversion device effectively mitigates intraluminal hypertension, prevents secondary injury, and improves therapeutic outcomes, thereby securing the critical physiological window required for definitive intervention. In terms of technical feasibility and short-term safety, no intraoperative or postoperative mortality was observed in either group, confirming the technical feasibility of the device in this controlled model. Although this 100% survival rate precludes a statistical comparison of mortality between groups, the successful application of the device establishes a new paradigm for managing abdominal combat trauma. Furthermore, its utility extends to civilian polytrauma scenarios (e.g., traffic accidents, natural disasters), demonstrating significant dual-use potential and broad translational prospects.

This study acknowledges several limitations regarding both experimental design and device configuration. First, the small sample size and the absence of direct intraluminal manometry or a standard enterostomy control group limit the generalizability of our findings. Subsequent adequately powered studies will address these constraints by incorporating catheter-based pressure monitoring, head-to-head comparisons with standard-of-care stoma strategies, and quantitative functional endpoints (e.g., motility, inflammatory markers) to rigorously validate clinical utility. Second, regarding device design, the current free-floating silicone prototype presents a potential risk of torsion and precludes the definitive isolation of material-specific effects from procedural benefits. To mitigate this, future iterations will integrate suture eyelets to ensure secure parietal fixation. Concurrently, we plan to conduct *in vitro* experiments and comparative studies of diverse biomaterials to further elucidate the underlying mechanisms of host-material interactions.

In summary, the temporary intestinal diversion device provides a new approach for the treatment of abdominal combat trauma. It is suitable for various types of intestinal injuries, and its application in the management of abdominal combat trauma represents a feasible and promising method with strong potential for future use.

## Data Availability

The original contributions presented in the study are included in the article/supplementary material, further inquiries can be directed to the corresponding authors.
